# INCREASING CONNECTIONS BETWEEN PERSONS WITH DEMENTIA AND CARE TEAMS: MY LIFE, MY STORY

**DOI:** 10.1093/geroni/igae098.1520

**Published:** 2024-12-31

**Authors:** Megan Gately, Jaye McLaren

**Affiliations:** VA Bedford Healthcare System, Bedford, Massachusetts, United States; Department of Veterans Affairs, Bedford, Massachusetts, United States

## Abstract

My Life, My Story (MLMS) was developed by the Veterans Health Administration (VHA) Office of Patient-Centered Care and Cultural Transformation as an opportunity for patients to share life stories with their care teams. Participating in MLMS interviews increases connection with clinicians, thereby facilitating patient-centered care. This study examines remote delivery of MLMS by occupational therapy students to patients of outpatient dementia clinics and their caregivers. Interviews were conducted using either videoconferencing, phone, or a combination. Post-interview questionnaires gathered Veteran-caregiver technological experience, including the need for technical support, comfort with technology, and how well they could see and hear. Questionnaires also gathered overall interview experience, including the belief that having care teams read stories would assist them in providing better care. In this session, we describe the MLMS program and objectives and present findings such as visit details (e.g., devices utilized, technological challenges, and caregiver role), and Veteran experience. We will also discuss strategies for conducting virtual interviews in the presence of cognitive concerns, such as interviewer flexibility and adaptability around technology, and communication strategies like simplifying questions and allowing extra time to accommodate for technology lag. Virtual narrative interviews using a structured approach like My Life, My Story allow for increased connection between individuals living with dementia and the care team, thereby facilitating patient-centered care. By providing an opportunity for social engagement, MLMS interviews may also address the growing scourge of older adult loneliness and social isolation, which is exacerbated for persons living with dementia.

